# Outcomes of Surgery in Patients with Lumbar Spinal Canal Stenosis: Comparison of Three Types of Stenosis on MRI

**DOI:** 10.1371/journal.pone.0158041

**Published:** 2016-06-22

**Authors:** Parisa Azimi, Shirzad Azhari, Edward C. Benzel, Hamid Khayat Kashany, Hossein Nayeb Aghaei, Hassan Reza Mohammadi, Meysam Ebrahimi

**Affiliations:** 1 Functional Neurosurgery Research Center, Shahid Beheshti University of Medical Sciences, Tehran, Iran; 2 Cleveland Clinic Foundation, Department of Neurosurgery, Cleveland, Ohio, United States of America; Universita degli Studi di Palermo, ITALY

## Abstract

The aim of the study was to compare outcome of surgery in patients with lumbar canal stenosis (LCS) based on magnetic resonance imaging (MRI) morphology. This was a prospective study of 96 consecutive patients who underwent surgery at 143 levels of LCS (from L3-L4 to L5-S1). Using patterns on T2 axial MRI, the type of stenosis was determined for each patient. The Swiss Spinal Stenosis Score (SSS) was used to evaluate patients’ functionality and outcomes. The definition of treatment success was based on the criteria developed by Stucki et al. Demographic characteristics and post-operative outcomes were compared between trefoil, triangular, and pin-hole groups. Finally, correlation between SSS score and the MRI morphology was assessed. The mean age of patients was 58.4 (SD = 8.9) years. Post-treatment satisfaction was observed in a large portion of the patients (87.5%). The trefoil group (n = 44), triangular group (n = 38), and pin-hole group (n = 14) had similar pre-operative Swiss Spinal Stenosis Score and were not significantly different in age, operative time, blood loss, duration of symptoms, walking distance, symptom severity and physical function (all p>0.4). No correlation between SSS score and the MRI morphology was observed. The findings suggest that the type of stenosis based on magnetic resonance imaging morphology is not indicative of surgical outcome among lumbar canal stenosis patients who undergo surgery at 1-year follow-up.

## Introduction

Lumbar canal stenosis (LCS) is associated with degeneration of the spine with aging. It is related to reduced space available for the neural and vascular elements of the lumbar spine. Patients can develop back pain as well as pain, weakness, and numbness or decreased sensation in the legs. It is the most common reason for spinal surgery in patients over 65 years [1, 2]. Although, surgical intervention may be effective for lumbar canal stenosis, there is a lack of consensus regarding indications for LCS surgery among surgeons. In addition there is insufficient evidence to guide clinical practice [1,3].

Clear unified radiological signs diagnostic criteria for decision–making process do not exist for patients with lumbar canal stenosis [4]. Moreover, none of the quantitative parameters measured on imaging studies correlate well with severity of clinical symptoms in lumbar canal stenosis [5–9].

At present, magnetic resonance imaging (MRI) is the most commonly used imaging modality for diagnosing LSS [10]. Recently, Memon et al. defined patterns on T2 axial MRI images that were used to define type of stenosis [9]. The type of stenosis, based on magnetic resonance imaging morphology, may represent a feasible method to morphologically characterize in patients with lumbar canal stenosis and it may help for following up the patients after treatment [9]. It was reported that further prospective studies were needed to determine the outcome of treatment based on magnetic resonance imaging morphology [9]. In addition, for surgical outcomes, the question regarding whether or not type of stenosis affects surgical result is yet to be answered. Hence, the purpose of the present study was to compare health related quality of life and satisfaction scores (before and) after surgery for patients with lumbar canal stenosis based on magnetic resonance imaging morphology at 1-year follow-up based on Swiss Spinal Stenosis Score (SSS). Moreover, patients’ functionality before surgery was also compared among the lumbar canal stenosis patients groups according to the SSS score. Finally, correlation between the SSS score and the magnetic resonance imaging morphology stenotic grade also was assessed.

## Methods

### Patients and data collection

A total of 96 consecutive patients (out of 111 who fulfilled the inclusion criteria) who were operated at 143 levels of LCS (from L3-L4 to L5-S1) were reviewed prospectively, between November 2013 and November 2014 in two teaching hospitals in Tehran, Iran. All patients had the typical symptoms of lumbar canal stenosis, such as neurogenic intermittent claudication and leg pain and/or numbness. The diagnosis of LCS was established by neurological examinations, clinical symptoms, and imaging studies including plain radiography, magnetic resonance imaging and computed tomography of the lumbar spine. More than one spine surgeon confirmed the diagnosis. All patients received conservative treatment at least for 6-months [11]. Patients received surgery (laminectomy without fusion at one to three levels, unilaterally or bilaterally, depending on the degree of stenosis) if conservative treatment failed. Using patterns on T2 axial MRI, the type of stenosis was determined for each patient. Types of axial image features were identified in lumbar canal stenosis to be symmetrical and asymmetrical with 5 subtypes. The morphologic types were labeled as trefoil, triangular, “cat’s eye,” “pin-hole,” and “no-hole” varieties as defined by Menon et al [9]. In the trefoil type, there were 3 subtypes A, B, and C. The triangular type has 2 subtypes: large and small. The large triangle is an isosceles triangle elongated in the antero-posterior (AP) direction and the disk is largely noncontributing to the canal compromise. In the small triangle, the shape is more equilateral and the AP dimensions are also significantly diminished [9]. The asymmetrical trefoil and triangular patterns were defined by Menon et al [9]. MRI morphology was measured at the maximal stenosis level. Clinical information including age, body weight (Kg), duration of symptoms (months), walking distance (m), blood loss (cc), operative time (min), the level of stenotic and types of lumbar canal stenosis were assessed. There were no limitations on patient selection with regard to types of LCS, age or other characteristics. We excluded all patients with prior lumbar spine surgery and spinal anomalies from the study. Moreover, regarding the population considered, were patients with spondylolisthesis excluded from this study. All patients underwent clinical evaluations pre-operatively and at 1-year postoperatively. Surgical outcomes were analyzed based on the SSS score. Based on at least 25% failure rate for surgery we estimated that a sample of 118 patients would be enough to have a study of 80% power at 5% significant level. However, we recruited 111 patients for the study.

### Additional measure

The Swiss Spinal Stenosis Score (SSS): The SSS has three domains: the severity of symptoms, physical functioning and patient’s satisfaction after treatment. It consisted of 18 questions. There are 12 questions for all patients, and a further 6 questions for those who have had treatment. The symptom severity scale (questions 1 to 7): Possible range of the score is 1 to 5.; the physical function scale (questions 8 to 12): Possible range of scores is 1 to 4.; and the satisfaction (with treatment) scale (questions 13 to 18): the range of the scale is 1 to 4. ‘‘1” represents the best possible score, whereas ‘‘5” and ‘‘4” represents the worst possible score. The subscale score is calculated by summation of all the scores of items. The score increases with worsening disability [12].

### Successful outcome measure

Patient satisfaction was considered as an outcome measure in order to indicate whether patients were satisfied with treatment they received at last follow-up. We used a standard questionnaire (questions 13–18 of SSS) for measuring satisfaction [12–13]. A mean score of 2.5 or lower was considered as successful outcome based on the criteria presented by Stucki et al [13]. The reference points for this study were the date of the initial surgery. The primary end points for the statistical analysis were 1 year of follow-up.

### Statistical analysis

Descriptive statistics such as means, standard deviations and percentages were used to explore the quantitative and categorical study variables. One-way analysis of variance (abbreviated one-way ANOVA) followed by Bonferroni’s post hoc comparison was applied to compare the means of all quantitative variables in relation to the trefoil group, triangular group and pin-hole groups. Student’s t-testing for continuous data and χ 2 tests for categorical data was used while statistical significance level was defined as p < 0.05. The data from patients who had been lost to follow-up were considered censored observations. The SPSS version 18 software package (SPSS Inc., Chicago, IL, USA) was used for all analyses.

### Ethics

Each participant gave informed verbal consent. Since some patients were less educated, for consistency we only asked for verbal consent. The main investigator explained the study for each participant and asked for permission. It was indicated that participation and no participation does not influence the treatment and their information will remain confidential. The Ethics Committee of Shahid Beheshti University of Medical Sciences, Tehran, Iran, approved the study and agreed with the consent procedure.

## Results

In all out of 111 patients, 96 patients were included in this study. Overall, there were 15 patients who dropped out of the study to prevent bias “cat’s eye,” group (n = 4), “indefinable shapes” (n = 3) group and “complete obliteration” group (n = 3), or who were lost at follow-up (n = 5). These patients were, hence excluded from the analysis. The mean age of patients evaluated was 58.4 years (SD = 8.9) (range, 34–84 years). The mean follow-up time was 12.3 months (ranging from 12 to 13). The characteristics of the lumbar canal stenosis patients and their MRI morphology, as well as the SSS scores, are shown in Table 1. Most patients (85.4%) had a type of trefoil and triangular stenosis. The trefoil group (n = 44; symmetrical, n = 29), triangular group (n = 38; symmetrical, n = 27), and pin-hole group (n = 14) had similar pre-operative SSS scores and were not significantly different in age, symptom duration, or follow-up periods (P > 0.4 for all). To compare patient's characteristics with three types of stenosis groups, one-way ANOVA followed by *post hoc* comparison of multiple variables by Bonferroni’s method was performed. All the parameters studied, had not a significant correlation with stenosis grading [Table 1]. In addition, post hoc ANOVA were not showed a significant difference between the both groups (trefoil vs triangular; trefoil vs pin-hole and triangular vs pin-hole stenosis) (P > 0.05 for all). Meanwhile, there was no correlation between SSS score (symptom severity and physical function) and the radiologic stenotic grade in the present study.

**Table 1:** Baseline demographic data and health status measures based on type of stenosis on MRI in patients with lumbar spinal canal stenosis (n = 96).

	Patterns of Lumbar Canal Stenosis*			
	Trefoil (n = 44)	Triangle (n = 38)	Pin-hole (n = 14)	
				ANOVA^#^
Characteristics				*P-*value^##^
Age (Year)	58.7 (7.9)	57.9 (9.6)	59.1 (9.8)	0.794
*Range*	40 to 82	34 to 84	41 to 82	
Gender (Male %)	52.3	50	50	0.714
Body weight (kg)	81.1 (9.3)	82.4 (9.7)	83.4 (9.5)	0.854
**Operative time (min)**	135.7(20.3)	133.3(19.1)	141.7(20.4)	0.524
**Blood loss(cc)**	270.3 (29)	264.7 (34)	278.3 (38)	0.345
**Lumbar stenosis levels**				
One-level, n (%)	25(56.8)	22(57.9)	8(57.1)	0.748
Two-level, n (%)	16(36.4)	14(36.8)	5(35.8)	0.864
Three-level, n (%)	3(6.8)	2(5.3)	1(7.1)	0.894
**Symptoms**				
Duration of symptoms (months)	41.6 (20.2)	37.2 (19.1)	36.9 (20.4)	0.653
Walking distance (m)	327.3 (254)	351.7 (280)	338.8 (261)	0.467
**SSS score**^¶^				
Symptom severity Q1–Q7	3.44 (0.31)	3.31 (0.48)	3.35 (0.34)	0.625
Physical function Q8–Q12	2.68 (0.32)	2.51 (0.53)	2.58 (0.38)	0.721
**SSS score**^¶¶^				
SSS Q13–Q18 (mean score, SD)	2.06 (0.42)	1.98 (0.46)	2.02 (0.43)	0.893

Values are mean (SD) or number (%).

* MRI Morphology: Using patterns on T2 axial MRI, the type of stenosis was determined for each patient. Types of axial image features were identified in LCS symmetrical and asymmetrical with 5 subtypes. The morphologic types were labeled as trefoil, triangular, “cat’s eye,” “pin-hole,” and “no hole” varieties as defined by Menon et al [8].

^¶^ The Swiss Spinal Stenosis Score, higher scores indicate worsening disability

^**¶ ¶**^A mean score of 2.5 or lower for SSS Q13–Q18 was considered as successful outcome.

^#^ Derived from one way analysis of variance (abbreviated one-way ANOVA).

^##^ Post hoc ANOVA analysis were not showed a significant difference between the both groups (trefoil *vs* triangular; trefoil *vs* pin-hole and triangular *vs* pin-hole stenosis) (P > 0.05 for all).

A mean score of 2.5 or lower for SSS Q13–Q18 was considered as successful outcome. Based on type of stenosis surgery successful outcomes are shown in Table 2. It was found that 87.5% (n = 84) of patients had surgical successful outcome at 1-year follow-up. No significant differences between groups were found, based on type of stenosis.

**Table 2:** Outcomes by type of stenosis based on SSS Q13–Q18 score in patients with lumbar spinal canal stenosis (n = 96).

	Successful (n = 84) *	Not successful (n = 12)	
Patterns of Lumbar Canal Stenosis**			P-value
*Trefoil*			<0.001
Type A, n (%)	5 (83.3)	1 (16.7)	
Type B, n (%)	19 (86.4)	3 (13.6)	
Type C, n (%)	14 (87.5)	2 (12.5)	
Total	38(86.4)	6(13.6)	
*Triangle*			<0.001
Type A, n (%)	16(88.8)	2 (11.2)	
Type B, n (%)	18(90.0)	2 (10.0)	
Total	34(89.5)	4(10.5)	
*Pin-hole*	12(85.7)	2(14.3)	<0.001
*Total*	84(87.5)	12(12.5)	<0.001

*A mean score of 2.5 or lower for SSS Q13–Q18 was considered as successful outcome.

** MRI Morphology: Using patterns on T2 axial MRI, the type of stenosis was determined for each patient. Types of axial image features were identified in LCS symmetrical and asymmetrical with 5 subtypes. The morphologic types were labeled as trefoil, triangular, “cat’s eye,” “pin-hole,” and “no hole” varieties as defined by Menon et al [8].

Intra-operative dural tears were recorded in 8 cases (trefoil (n = 4), triangle (n = 3), pin-hole (n = 1)) and were repaired. No difference in operative time and blood loss were observed between three groups (Table 1).

## Discussion

Our results showed that no differences in surgical outcomes were observed between the magnetic resonance imaging morphology types in patients with lumbar canal stenosis at 1-year follow-up. Hence, the type of stenosis may not be a pre-operative predictor of surgical success.

Many investigators have sought to identify the pre-operative variables that predict a successful outcome following lumbar canal stenosis; however, the results obtained were often divergent [1, 14]. These discrepancies between studies are probably due to the different ages of the populations studied and the varying lengths of follow-up. Kim et al. [14], reported that motor weakness, the subjective amount of disability of daily activity, and Schizas grades C and D [5], were associated with a higher odds of a surgical decision. Moreover, they also demonstrated that women with lumbar canal stenosis were more likely to opt for conservative treatment than men [14]. Other studies and our previous finding [5, 14–15] demonstrated that patients with a severe stenosis (C and D) group had benefits from surgery. In this study, we investigated the differences in morphology grade before surgery between the groups. Although we observed good surgical outcomes after surgery, the type of stenosis was not shown to be a predictor of surgical success.

Prior to this study, to our knowledge, there have been no investigations to determine pre-operative predictor of surgical success based on MRI morphology in patients with lumbar canal stenosis. For assessing the severity of LCS, a semi-quantitative grading of stenosis was used according to dural sac morphology and the relation of cerebrospinal fluid (CSF) to nerve roots in the lumbar canal [5, 16]. These grading schemas demonstrated initial promise as success predicting factors [5, 16]. However, a newer morphology grading scheme was presented by Menon et al. [9]. They reported that the pin-hole group represented the more severe stenosis. However, in our study, this was not observed this issue based on the SSS score compared to other two groups. This difference may potentially be associated with the small subset (pin-hole group) of the study population in which we were unable to show the reason for this effect. Meanwhile, in our series, the magnetic resonance imaging morphology was measured at the maximal stenosis level. However, in the study by Menon et al. the magnetic resonance imaging morphology was considered at all levels of stenosis. Consequently, a standardization of evaluation of the significance of imaging parameters according to treatment outcomes should be studied [17].

Most research to date has not shown an association between clinical symptoms and findings on imaging [1, 18–19], which was consistent with our results. The lack of association might partly be due to the procedure used in imaging, as the patient is supine at the time of imaging [1], while symptom severity and physical function in patients with lumbar canal stenosis usually presents during standing or walking

There are several principle weaknesses of this study. First, there is a lack of standard criteria for diagnosis and for inclusion in the study. In fact we suspect that there might be inter and itera-observer bias in diagnosis. Second, in this study, treatment success was based on SSS score. This may have resulted in a selection bias. However, the definition of a surgical success outcome depends entirely on the criteria used. Third, no statistical analysis was performed to compare between symmetrical and asymmetrical in groups due to a suboptimal number of cases. Fourth, multiple grades of stenosis co-existed was not evaluated in the same lumbar spine. Fifth, the patient income was not assessed, which can influence treatment decision-making. Perhaps those with higher income might be chosen better treatment options and thus achieved better outcomes. Sixth, we were unable to assess all medical interventions and related complications. Hence, further studies with such data are needed. Finally, surgery outcome in the present study was recorded only 1-year post-operatively and only three types of stenosis were studied. Future studies might examine predictive utility at longer follow-up intervals and other types of MRI morphology.

## Conclusion

There was no difference in surgical outcomes according to magnetic resonance imaging morphology among the lumbar canal stenosis patients who underwent surgery at 1-year follow-up. Further analysis, comprised of a larger, longitudinal sample, would contribute to outcomes research, and assist with future practice guideline development.

## Supporting Information

S1 File A minimal set of data for the study. (SAV)
